# 

**Published:** 1891-12

**Authors:** 


DECEMBER, 1891.

*Vol. IX.* *No.* **34.**

THE

### BRISTOL MEDICO-CHIRURGICAL JOURNAL

A Quarterly Journal of the Medical Sciences for the West of England and South Wales

*PUBLISHED UNDER THE AUSPICES OF*
*THE BRISTOL MEDICO-CHIRURGICAL SOCIETY.*

LIBRARY OF CONGRESS,
MAY 10 1897
SMITHSONIAN DEPOSIT.

EDITOR:

J. GREIG SMITH, M.A., F.R.S.E.

ASSOCIATED WITH

BARCLAY J. BARON, M.B., C.M.

J. MICHELL CLARKE, M.B., M.A.

F. R. CROSS, M.B.

W. H. HARSANT.

ASSISTANT-EDITOR:
L. M. GRIFFITHS.

THE LIBRARY OF THE BRISTOL MEDICO-CHIRURGICAL SOCIETY.

CANCELLED
Date 27/7/97
Sig.

"Scire est nescire, nisi id me

Scire alius sciret."

BRISTOL: J. W. ARROWSMITH;
LONDON: J. & A. CHURCHILL, 11 NEW BURLINGTON STREET.

*Price One Shilling and Sixpence.*

